# Medical Opinion and Movement

**Published:** 1910-03-26

**Authors:** 


### Medical Opinion and Movement.

### Cancer in Mice.

Under the direction of Dr. Borel, Dr. Nègre has carried out some interesting research on the etiology of cancer in mice. He has observed that mice affected with adenosarcoma of the mammæ are also frequently infected with spirillosis, and he has attested the presence of spirillæ in malignant tumours of mice. He has therefore endeavoured to ascertain the possible etiological connection between the two maladies by inoculating a large number of mice with spirillæ. Among 234 mice so inoculated ten developed tumours spontaneously, whereas of 392 control mice only two developed tumours. From these facts he concludes that spirillosis is a predisposing cause to the development of cancer in mice. He finds, further, that the sites of the tumours are for the most part those regions where the parasites tend to accumulate. The predominance of the tumours at spring and autumn also corresponds to the time of sporulation of the parasites. Experimenting further with cancer inoculations, he finds that by feeding the mice with large quantities of chlorine salts—sodium, potassium, barium, and calcium chloride—the animals are rendered more impervious to cancer inoculations. It appears that the cancer growths can be acclimatised to these salts, so that if an animal already affected with cancer be saturated with one of these salts, inoculation of the growth into another animal that has previously been fed with the same salt will succeed. Such inoculations are, however, less successful with barium or sodium chloride than with the other chlorides. It would appear, then, that these chlorides are especially deterrents to the development of cancer.

### Tendinous Reflexes in Diagnosis.

It is a recognised fact that the patellary reflex may be very feeble or absent in otherwise healthy individuals, or that it may be temporarily absent in functional and hysterical conditions. According to Dr. Böttiger the definite significance of such absence may be determined by passing a triphasic alternating current through the limbs for a short time. During and for some time after such stimulation the reflexes, both patellar and Achillean, will return if their absence is of a functional nature, but in cases of organic disease—tabes dorsalis and the like—excitation by the current has no effect. To apply the test, the patient is seated, the feet bared, and an electrode from a triphasic alternating current is applied to each foot, and the third electrode is grasped by the hands. The strength of the current is gradually increased, taking care to stop before there is dorsal flexion of the toes and fingers. The reflexes are then sought for in the usual way. Dr. Böttiger supposes that the electric current acts by increasing the muscular tone. He reports several cases in which the method has been of assistance in diagnosis. A railway *employé* was affected with double facial paralysis, with the Achillean reflexes markedly diminished. The railway doctors diagnosed syphilitic encephalitis, in spite of denials on the part of the patient, and he was dismissed without pension. Dr. Böttiger was able to show by means of his method that the diminution in the reflexes was functional, and that the facial paralysis was of a rheumatic nature.

### Influenza and the Pneumococcus.

About a year ago Dr. Curschmann described an influenza epidemic at Leipzig during the winter 1907-8, in which the causal agent appeared to be the pneumococcus. More recently Dr. Rose has reported in the *Münchner Medizinische Wochenschrift* a similar epidemic in the Municipal Hospital of Strassburg, in which 37 out of 84 inmates were affected, and 16 out of 26 of the *personnel*. The course and symptoms of the malady resembled influenza, but bacteriological examination always showed the presence of the diplococcus of Fränkel-Weichselbaum. The incubation period was usually 12 to 36 hours, occasionally two or three days. The disease commenced with a sudden rise of temperature, accompanied by a rigor, and there were the usual headache and pains in the back and limbs. In a few cases the local signs amounted to a more or less intense pharyngitis, but in eight cases there was bronchitis or broncho-pneumonia, and in 17 cases a fibrinous pneumonia. Other complications were: purulent otitis, one case; purulent arthritis of knee, one case; hæmorrhagic nephritis, three cases; and severe diarrhœa, three cases. The fever was remittent, and generally lasted 10 to 12 days, and ended by lysis. In the cases of pneumonia the fever lasted three or four weeks, and terminated by lysis. There were seven deaths, all among the cases of pneumonia. The pneumococcus was found in the expectoration and other discharges, generally in pure culture, but occasionally associated with Friedländer's bacillus and with the micrococcus catarrhalis. Examination of the blood showed the presence of the pneumococcus in only two cases. Both ended fatally. In no case was the bacillus of Pfeifer found present. In this connection it is worthy of record that during this winter, in the experience of several medical men here, similar cases have been observed, in which, together with symptoms in all respects identical with those of influenza, the fever has taken a protracted course and has failed to yield rapidly to the usual medical treatment. There has been a troublesome cough, and the signs of the chest have shown either the presence of a bronchitis or definite patches of pneumonia, usually apical, and bacteriological examination of the sputum has revealed the presence of the pneumococcus. The fever is of a remittent type, and, as in the epidemic at Strassburg, terminates by lysis.

### The Effects of Abdominal Impalement.

Dr. Pauchet, of Amiens, at a meeting of the Société de Chirurgie, reported the case of a girl of eight whom he was called upon to attend as the result of impalement on a spiked railing. The spike, after tearing the perineum so that rectum and vagina opened into a common passage, entered the rectum and penetrated thence into the abdominal cavity. The consequent shock was treated by subcutaneous injections of saline fluid and antitetanic serum. The following day, owing to high temperature, rapid pulse, tympanites, and persistent vomiting, laparotomy was performed. The peritoneum above the level of the umbilicus was found to be healthy, but peritonitis was found below that level. The rectum was seen to be pierced at the level of the pouch of Douglas, and the pelvic colon torn and crushed against the sacral promontory. The latter tear was sutured, but it was found impossible to suture the rectum owing to the lacerated condition of the organ. A large tube was, therefore, inserted through the wound into Douglas's pouch, and kept in place by stitching its upper end to the posterior surface of the uterus. The pelvis was isolated from the general cavity by suturing the pelvic colon to the bladder as after a panhysterectomy. No attempt was made to repair the perineum, but a left iliac colotomy was performed. The abdomen was then closed. For forty-eight hours after operation the patient passed no flatus, and a second operation, enterostomy, was performed. This was followed by collapse, general infection, and a temperature of 104° F. Cold baths brought about an immediate improvement, the temperature quickly regaining and retaining the normal height. Ten days later the tube was removed. A month after the accident, at a second operation, the continuity of the bowel was restored and the iliac wound closed. At the same time the perineum was repaired. This operation was followed by high temperature, a running pulse, and signs of a septicæmic condition, due no doubt to infection of the uterus at the time of the accident which had been unable to drain properly owing to the small size of the vulva. Cold baths administered twice led to a speedy convalescence. The author thought the case worthy of note (1) because it showed the nature of the injuries following impalement, (2) because of the efficacy of enterostomy in relieving paralytic conditions of the intestine in peritonitis, and (3) because of the favourable influence of cold baths in grave surgical infections.

### Korsakow Syndrome.

The relations of alcoholism to insanity are numerous and intricate, but that in which the former seems most definitely and certainly the causative agent of the latter is the not very uncommon, though often undiagnosed, disease known as Korsakow's Polyneuritic Psychosis. In the *Journal of Mental Science* Dr. J. Turner supports forcibly the claims of Korsakow regarding the distinctive entity of this disease, claims which have in this country been defended especially by Ascherson. Moreover, he believes that it is only when the Korsakow syndrome is exhibited that it is justifiable to assign alcoholism as the prime cause of the insanity. This position is one not generally allowed, and many authorities even contend that the polyneuritic psychosis in question need not necessarily be caused by alcoholism. Dr. Turner, of course, recognises that alcohol is frequently associated with many forms of insanity, other than Korsakow's disease; but he holds that the mental deficiency and the over-indulgence are merely both sequential upon defective inhibitory powers; and that whenever alcoholism is the sole antecedent of insanity, then that insanity is of the type under discussion. Even delirium tremens is held to be probably not directly an effect of alcohol itself, but more likely the result of the accumulation of some toxin or toxins not directly alcoholic; but several observers have tried to see in this an acute form of the much more chronic condition.

The essential feature of Korsakow's disease is neuritis in some part or other of the nervous system. The mental condition may be at first unclouded, but the nerve cell branches fail to convey completely the impulses originating in the nerve cell region: there may be thickness of speech, incoordination of gait, blunting of sensation, muscular hyperæsthesia, and other evidence of neuritic involvement at a time when the psychical functions may be apparently perfect. There is said to be a characteristic physiognomy, apart altogether from capillary injection, acne rosacea, and so on. The general expression is described as combining silly self-satisfaction with a look of astonishment: there may also be a very rapid fine tremor of the lip, not always visible to the eye, but evident photographically, and slight ptosis may be present. The deep reflexes are nearly always deficient or absent: a case of insanity with absent knee-jerks, but without the Argyll-Robertson pupil, should always raise suspicion of alcoholic origin. The pupils are often sluggish to light, but never inactive altogether. Mentally, the three cardinal symptoms are loss of memory, disorientation of place, time, and persons, and pseudo-reminiscence. Loss of memory is chiefly for recent events, and is a variable factor from day to day: at one time the patient can remember things fairly well, at another he will forget something five minutes after being told it. Total amnesia is not found. Disorientation is equally common, and, like the last-mentioned, is always found at some time or another in the course of each case. Pseudo-reminiscence is less constant; this is the name given to the kind of delusion in which the patient gives a long account of imaginary doings which have never taken place at all. The author concludes with an elaborate research into the histopathology of the nervous system in this disease.

### Midwifery Forceps Operations.

In an article dealing with the preparation and position of, and other points in connection with, patients to be delivered by the use of midwifery forceps, Dr. McDonald, of New York, publishes in the *American Journal of Obstetrics* a description of a pattern of forceps which seems to be quite different from those commonly used. The pattern he recommends is described as a modification of the Tucker-McLane pattern. The blades are not fenestrated as in the usual patterns, but are practically solid, with about a dozen narrow slit-like fenestræ set parallel and across their length. The forceps is especially constructed so that the shanks remain quite narrow whether the blades are completely or only partially closed; and the total length of the instrument is a good deal less than in the Simpson type. When the forceps is closed, there is 3.5 c.m. between the tips of the blades and 7.5 c.m. between the widest parts. The (arc of the) curve is 13.5 c.m. in length, and the total length over all is 39 c.m. When the biparietal diameter of an average head is held in the blades, the tips are separated by 5.5 c.m. The blades are much narrower than in the usual British patterns. It is claimed that these forceps fit the fœtal head better than others, that they take up less room in the pelvis, and are less liable to cause lacerations, that they are less likely to slip off, and that there is less chance of damaging the head or neck of the fœtus with them.

---

**Pneumococcus Vaccine.**

It would seem probable from the claims of several bacteriologists that the armamentarium of the physician in dealing with pneumonia is to be strengthened very considerably by the inclusion of vaccine methods. The difficulty of estimating accurately the influence of any new factor in treatment upon this or other acute and severe disease of which the average prognosis is nevertheless good, is that it is impossible or at least very difficult to disentangle the successes of the method from the natural cure due to the patient's own reaction and recuperation. A small series of cases, published in the *Medical Record*, by Dr. H. A. Craig, is of especial interest in this respect, because, although they are but six in number, the ages of the patients ranged from 66 to 83. Dr. Craig's plan is to give at once a dose of 20 to 35 killed pneumococci (stock) and to prepare as soon as may be further doses from the organism isolated from the patient's own sputum; fifty millions is the maximum dose he gives. The most striking of the six cases is that of a man aged eighty-three, seen on the third day of his illness, who had been on a prolonged spree and was verging on delirium tremens; his general condition was very bad, he was delirious, and he had an extensive and severe pneumonia with albumin and casts in the urine. He had two injections of pneumococci, a crisis on the sixth day, and was out of bed on the eighth day; was discharged well on the eleventh day, and has remained well since.

---

**Treatment of Epistaxis.**

In the *Australasian Medical Gazette* Mr. James Boyd describes a simple method of plugging the nares for obstinate epistaxis which has resisted the usual routine of compression, cold or iced douching, smart purgation, and anterior plugging. He very truly remarks that the operation of posterior plugging as commonly performed is very unpleasant to the patient, and that it requires special instruments which are nine times out of ten not procurable in an emergency. The plan he recommends is easily and quickly followed, and requires only strips of clean muslin and a pair of dressing forceps; it is said to be almost as efficient as Rose's bag, and hardly more than inconvenient to both patient and operator. A dry piece of fine-starched muslin about six inches square is wrapped round the points of a closed pair of dressing forceps, by placing the latter in the centre of the square and then folding the muslin round the blades umbrella-fashion. This is then passed through the nares until it impinges on the posterior pharyngeal wall; the forceps is then withdrawn. The edges of the muslin are spread out over the face, and the hollow cone inside the nose is rapidly plugged with small pieces of cotton wool soaked in any available styptic-vinegar in default of the more usual astringent drugs. If—says the author—it is not necessary to plug the post nasal fossa, the muslin can be pulled very slightly forwards after the withdrawal of the forceps, so as to clear the posterior wall of pressure before the plugs are introduced. This is worth bearing in mind, as it avoids the temporary deafness otherwise set up.

---

**Fatality under Spinal Anæsthesia.**

By this time a large number of accidents have been put on record as the result of lumbar anæsthesia, and probably there have been at least as many, if not more, unpublished. In the *Indian Medical Gazette* Major Gabbett describes an interesting case in which death followed the insertion through the eleventh dorsal interspace of one milligramme of strychnine hydrochloride with one decigramme of novocain. It is difficult to decide from the account whether the fatal issue arose from strychnine poisoning or from overdose of novocain; it seems at least certain that respiratory failure was the direct cause of it. The patient was a native, aged forty-one, who was to have a large elephantiasis of the scrotum removed, weighing 31 lb. Five minutes after the spinal injection he had anæsthesia up to the nipples, and ten minutes later still it had reached three inches above that level. He sat up for one minute after receiving the dose, and then lay down with his head on a pillow. Half an hour after the injection, just before the completion of the operation, he had dyspnœa and tried to vomit, and shortly after ceased breathing. All efforts at resuscitation failed, and it is mentioned that stiffness of the arms somewhat impeded artificial respiration. There had been practically no loss of blood and no shock, and the author attributes the result to spasm from the action of the strychnine rather than to the action of the novocain. He concludes by resolving to leave pioneer work alone in future, and to stick to general anæsthesia until spinal methods are improved by others.

